# What is Glycaemic Variability and which Pharmacological Treatment Options are Effective? A Narrative Review

**DOI:** 10.17925/EE.2023.19.2.4

**Published:** 2023-07-07

**Authors:** Juan Miguel Huertas Cañas, Maria Alejandra Gomez Gutierrez, Andres Bedoya Ossa

**Affiliations:** Pontificia Universidad Javeriana, Bogotá, Colombia

**Keywords:** Continuous glucose monitoring, diabetes mellitus, glycaemic variability, hyperglycaemia, hypoglycaemia

## Abstract

Glycated haemoglobin is currently used for diagnosis and follow-up of diabetes mellitus. However, it has important limitations; as it only reflects the average glycaemia over the last 3 months, it does not allow the identification of crucial events, such as episodes of hypoglycaemia and hyperglycaemia. Strict control of hyperglycaemia can result in severe hypoglycaemia that can be life threatening and can have important sequelae. Recently, the concept of glycaemic variability has been developed to provide information about the magnitude of glycaemic excursions and the duration of these fluctuations. This new approach has the potential to improve outcomes, decrease the risk of hypoglycaemia, and decrease cardiovascular risk. This review describes the most commonly prescribed non-insulin anti-diabetic drugs for diabetes management, their mechanism of action, and the existing evidence about their effectiveness in improving glycaemic variability and diabetes control.

The incidence of diabetes has increased in recent years, and advances in technology have allowed for multiple ways to predict the outcomes of patients with diabetes, and have improved quality of life and lowered morbidity and mortality.^1^ For decades, glycated haemoglobin (HbA1c) has been used as a marker of long-term glycaemic control, and its usefulness as a predictor of complications in type 2 diabetes mellitus (T2D) was established in 1993 in the Diabetes Control and Complications Trial (DCCT).^2,3^ In 1999, the UK Prospective Diabetes Study (UKPDS) confirmed that reducing exposure to hyperglycaemia through intensive treatment with insulin or oral antidiabetic drugs significantly reduced the incidence of complications in T2D.^4^ There is an important association between mortality and increased HbA1c; in the DCCT cohort, a 10% increase in HbA1c was associated with a 56% increase in mortality.^5^ Nevertheless, its use has considerable limitations, as it is an average of the behaviour of glycaemia over the last 3 months and does not highlight significant excursions. This implies that it has a low capacity to predict the risk of severe hypoglycaemia, which was demonstrated in the DCCT that concluded that only 8% of episodes can be predicted by parameters known at that time, including HbA1c.^6^ Additionally, significant glycaemic excursions, including those generated by aggressive treatment, affect cognitive function, quality of life, cardiovascular disease and all-cause mortality.^7,8^ This attempt to optimize treatment has been described as a trade-off between glycaemic control and iatrogenic hypoglycaemia.^9,10^ In this context, measuring glycaemic variability (GV) represents a way to achieve treatment goals while avoiding the risks associated with both severe hypoglycaemia and prolonged exposure to hyperglycaemia.^11^

GV is defined as the measure of fluctuations in glucose or other parameters of glycaemic homeostasis over a given period. Although GV was initially approximated based on self-monitoring glucose measurements, these values represented a limited profile of glycaemic behaviour.^11^ With the advent of technologies such as continuous glucose monitoring (CGM), it is possible to obtain a more complete record and measure blood glucose at 5-minute intervals and overnight.^12^ CGM has proven useful for optimizing treatment in patients with diabetes, with multiple options to measure it, including real-time flash and professional.^13,14^ A wide repertoire of methods is currently used to assess GV, representing in some cases its short-term or long-term behaviour. Short-term GV refers to glucose fluctuations during the day that are usually measured with a CGM system, from which data, including the standard deviation (SD) of the average glycaemia, the coefficient of variation (CV) and the mean amplitude of glycaemic excursions (MAGE), are obtained.^12^ Although there are more complex ways to assess short-term GV, they are rarely used in clinical practice.^12^ Long-term GV refers to fluctuations in blood glucose over months to years. This is based on serial measurements of Hb1Ac, fasting blood glucose (FBG) and/or post-prandial blood glucose, from which the SD and CV are calculated.^15,16^

GV has been linked to unfavourable outcomes in patients with diabetes, specifically regarding the pathophysiological implications of high GV.^16–19^ Glycaemia oscillations are associated with the microvascular and macrovascular complications frequently observed in patients with diabetes. Macrovascular complications include coronary artery disease, cerebrovascular disease and peripheral vascular disease, whereas microvascular complications include neuropathy, retinopathy, nephropathy and lower limb ischaemia.^20,21^ Multiple studies indicate that fluctuations in glycaemia generate oxidative stress and activate the inflammatory cascade, which is more deleterious than sustained hyperglycaemia in the long term^22–24^ and occurs rather early in the course of the disease.^25–27^ The mechanism responsible is suspected to be an increased activation of nicotinamide adenine dinucleotide phosphate (NADPH) oxidase resulting in excessive production of reactive oxygen species, oxidative DNA damage and decreased superoxide dismutase activity.^28^ Additionally, GV contributes to the inflammatory response, with evidence that increased GV results in increased expression of proinflammatory mediators, including interleukin-6 and tumour necrosis factor α, compared with sustained hyperglycaemia.^29^ Fluctuations in glycaemia also affect nitric oxide synthesis^30^ and may suppress endothelial protective mechanisms against glycotoxicity-induced damage.^31^ The aforementioned mechanisms and the increased risk for hypoglycaemia implicates GV as a key factor in the development and progression of most complications associated with T2D.^12,32^

Despite this, there are no clearly defined reference values for GV. Possibilities include SD, CV, interquartile range, MAGE, mean absolute relative difference (MARD) and mean inter-day risk range; these allow us to determine which patients have stable glycaemic control and which have labile control.^16^ Among these, CV stands out since there is a consensus that establishes normal values. CV correlates with the risk of hypoglycaemia and is clinically useful as an indicator of GV. GV is considered stable when the CV is <36%, whereas CV values >36% indicate a labile GV and predict a high probability of a severe hypoglycaemia episode in the next 6 months.^33^

Recently, the time in range (TIR) metric was identified, which is defined as the percentage of time in which blood glucose is in a given range.^20,34^ In 2017, the Advanced Technologies and Treatments for Diabetes group reached a consensus to standardize the use of CGM;^33^ before this, different ranges were used, affecting comparison between studies. In general terms, the consensus established the following goals: >70% of a day with blood glucose 70–180 mg/dL (3.9–10.0 mmol/L), <4% at <70 mg/dL (3.9 mmol/L), <1% at <54 mg/dL (3.0 mmol/L), <25% at >180 mg/dL (10.0 mmol/L) and <5% at >250 mg/dL (13.9 mmol/L).^35^ There are individualized goals according to age group, clinical profile and other variables, but these are not the subject of this article.

Considering the above, GV needs to be added to the tools for controlling T2D, making it imperative to gain a comprehensive understanding of the impact that existing pharmaceutical interventions have on GV.

## Materials and methods

This article is a narrative review in which a literature search of articles in English and Spanish was carried out using PubMed, Medline and Embase databases. The following search key was used: ((“glycemic variability”) AND (type 2 diabetes mellitus)) AND (metformin); ((“glycemic variability”) AND (type 2 diabetes mellitus)) AND (sulfonylurea compounds); ((“glycemic variability”) AND (type 2 diabetes mellitus)) AND (dipeptidyl peptidase four inhibitors); ((“glycemic variability”) AND (type 2 diabetes mellitus)) AND (“sodium-glucose transporter two inhibitors”); ((“glycemic variability”) AND (type 2 diabetes mellitus)) AND (glucagon-l ike peptide one agonist); ((voglibose) OR (acarbose)) OR (miglitol)) OR (alphaglucosidase inhibitors)) AND (type 2 diabetes mellitus)) AND (glycemic variability); ((“glycemic variability”) AND (type 2 diabetes mellitus)) AND (insulin).

Only articles that explicitly addressed GV as one of the study variables and evaluated the response to treatment in patients with T2D were included. Articles evaluating drugs of interest ranging from 2008 to 2022 and that used monotherapy or reference treatment (metformin, insulin) as comparison were included. Articles prior to this time period were taken into account to consider the historical process of diabetes treatment and follow-up. All articles referenced through the descriptors were analysed and coded independently by two investigators, who read each article at least twice. No patient data was used and no interventions were performed.

## Results and discussion

### Insulin

Insulin therapy is a cornerstone of diabetes management, particularly for patients with type 1 diabetes (T1D) and those with inadequately controlled T2D. Fast-acting insulin, such as lispro and aspart, are typically administered before meals to control post-prandial hyperglycaemia. Meanwhile, basal-l ike insulins, such as degludec and glargine, provide a more stable and prolonged glycaemic control by mimicking endogenous insulin secretion that occurs between meals and during overnight fasting. By reducing fluctuations in blood glucose levels, basal-l ike insulins help lower GV, which is a crucial factor in preventing the acute and chronic complications of diabetes.^20,36^ Moreover, new generation basal-l ike insulins have been associated with a lower risk of hypoglycaemia and nocturnal hypoglycaemia compared with older-generation insulins.^37^

Yang et al. compared degludec with glargine and concluded that degludec was superior to glargine in reducing FBG variability in both T1D and T2D; degludec had a longer TIR than glargine 100 units but not longer than glargine 300 units.^38^ For other indicators of blood glucose variation, including SD, MAGE, mean blood glucose (MBG) and CV, no significant differences were identified between degludec and glargine.^38^ Vrebalov et al. concluded that there was no statistically significant difference in GV between degludec 100 units and glargine 300 units on parameters such as SD, MBG and CV.^39^ Oe et al. compared degludec + liraglutide versus a dipeptidyl peptidase 4 (DPP4) inhibitor + basal insulin; MAGE significantly decreased from 74.9 mg/dL (95% CI,60.3–97.7; p<0.05) to 64.8 mg/dL (95% CI, 52.0–78.2; p<0.05) in the degludec + liraglutide group, resulting in a better control of GV compared with the DPP-4 + basal insulin group.^36^

Basal-l ike insulins are a valuable treatment option for patients who require intensive glycaemic control while minimizing the risk of hypoglycaemia. However, the choice of insulin therapy should be individualized to each patient, taking into account factors such as age and comorbidities, and should be accompanied by comprehensive diabetes education and regular monitoring.^40,41^

### Metformin

Metformin is one of the most prescribed anti-diabetic drugs, and is considered the ideal initial therapy for patients with T2D due to its low cost, safety profile with low risk of hypoglycaemia, and favourable evidence in cardiovascular protection.^42^ Metformin lowers serum glucose through various mechanisms such as reducing hepatic glucose production by inhibiting gluconeogenesis and glycogenolysis, improving insulin sensitivity in skeletal muscle, and reducing intestinal glucose absorption.^43,44^

Pistrosch et al. compared insulin glargine versus metformin as first-line treatments in 75 patients with newly diagnosed T2D, evaluating GV, microvascular function and β cell function.^45^ In patients in the glargine group, fasting glycaemia decreased and β cell function improved, established by the post-prandial pro-insulin/C-peptide ratio. Regarding GV, the group receiving insulin glargine had greater fluctuations in glycaemia, expressed as MAGE and SD, showing that metformin decreases GV compared with insulin glargine.^45^

González-Heredia et al. studied the effect of linagliptin versus metformin on GV in a group of 16 patients with impaired glucose tolerance, following both groups for 3 months.^46^ The linagliptin group showed better glycaemic control when assessed by oral glucose tolerance. In regards to GV, there was no significant difference between groups.^46^ Takahashi et al. studied a group of Japanese patients and compared the use of high-dose metformin (HMET) as monotherapy (1,500 mg) versus low-dose metformin (750 mg) in combination with linagliptin (LMET + DPP4), and excluded patients with insulin treatment.^47^ Both groups were assessed by CGM and no clinically significant episodes of hypoglycaemia were observed. There was a lower post-prandial area under the curve (AUC) (p=0.041) in post-prandial glycaemic excursions in the LMET + DPP4 group compared with the HMET group. No significant between-group difference was observed in MAGE or SD.^47^

Difficulties were encountered during the literature search regarding the effect of metformin on GV because most of the studies compared metformin in combination with another oral anti-diabetic drug and few studies evaluated monotherapy. Since metformin is the standard treatment in T2D, research that met the criteria outlined in the Materials and Methods was limited, partly due to the difficulty of comparing against placebo and due to the high prevalence of metformin as initial treatment.

### Dipeptidyl peptidase 4 inhibitors

DPP4 inhibitors are incretin derivatives that inhibit the enzyme that degrades glucagon-l ike peptide 1 (GLP-1), thereby increasing GLP-1 and gastric inhibitory peptide. This, in turn, increases β cells' sensitivity and suppresses glucagon secretion.^48^ The risk of hypoglycaemia with DPP4 inhibitors is low due to their mechanism of action being mediated by GLP-1,^48^ which also allows its use as combination therapy.^49^ Although DPP4 inhibitors increase GLP-1 concentration, there are no changes in gastric emptying or gastric accommodation, which is an important difference from GLP-1 analogues.^50^ Although the mechanism is unknown, it is likely that DPP4 inhibitors decrease incretin secretion due to negative feedback on neuroendocrine cells.^50^

Kim et al. studied the effects of vildagliptin on GV, oxidative stress and endothelial parameters (urinary 8-i so-prostaglandin F2α and flow-mediated endothelial dilatation) in patients with poorly controlled T2D on metformin monotherapy. Vildagliptin significantly improved glycaemic control and reduced GV reflected in the MAGE and mean of daily differences (MODD).^51^

Nishimura et al. compared the effect of trelagliptin and alogliptin on GV in 27 patients with T2D, finding a post-prandial decrease in the AUC of -31.2 mg/dL min (95% CI, -105.8 to +43.3) in the trelagliptin group, and an increase of +0.4 mg/dL min (95% CI, -0.4, + 1.1) in the alogliptin group on day 22. The AUC for glycaemia <70 mg/dL was 0 on day 28 for both groups.^52^

Butaeva et al. compared carbohydrate metabolism markers such as HbA1c, FBG and post-prandial serum glucose, and GV in 51 patients with poorly controlled T2D on metformin monotherapy. The markers were compared before and after adding sitagliptin 100 mg/day or gliclazide modified release 60 mg/day for 3 months.^53^ The sitagliptin + metformin group obtained better results than the gliclazide + metformin group in the aforementioned markers, highlighting a significant reduction in MAGE and SD, reflecting a decrease in GV.^53^

Kim et al. studied patients with poorly controlled T2D on metformin monotherapy who were assigned to either vildagliptin or pioglitazone for 16 weeks and using CGM.^54^ Although there was no reduction in oxidative stress markers, there was a statistically significant reduction in GV in patients who received vildagliptin (MAGE from 93.8 ± 38.0 mg/dL to 70.8 ± 19.2 mg/dL; p=0.046).^54^ Tan et al. evaluated the effect on GV of adding DPP4 inhibitors to premixed human insulin in patients with T2D, finding an increase in TIR and decreases in SD and MAGE.^55^

Difficulties were encountered during the literature search regarding the effect of DPP4 inhibitors on GV because no studies evaluated monotherapy versus placebo, and very few compared drugs within the same pharmacological group or with reference drugs for the treatment of T2D, such as metformin or insulin. DPP4 inhibitors have a satisfactory effect on GV with an adequate level of evidence.

### Sulphonylureas

Sulphonylureas close adenosine triphosphate-sensitive potassium channels, which eventually stimulates insulin release.^56^ First-generation sulphonylureas, such as tolbutamide, are associated with weight gain, severe hypoglycaemia, mortality, cardiovascular events and dementia.^57^ Newer extended-release sulphonylureas are safer due to the reduced risk of hypoglycaemia.^56^

Kohnert et al. studied 59 patients with T2D treated with metformin, sulphonylureas, a combination of these two, or lifestyle modifications.^58^ Sulphonylureas were associated with a longer time in hyperglycaemia (10.3 versus 0.9 h/day; p<0.001) when compared with diet alone, with no significant differences regarding hypoglycaemia. The pooled sulphonylurea and metformin group had a higher MAGE (5.7 mmol/L versus 3.6 mmol/L; p≤0.001) when compared with lifestyle modifications alone.^58^ On the other hand, Uemura et al. evaluated 123 hospitalized patients with a TIR >70% to determine the risk of hypoglycaemia in patients with apparent good metabolic control, with or without the use of sulphonylureas.^59^ An association was observed between the use of sulphonylureas and greater GV, increasing the %CV by 2.678 (95% CI, 0.211–5.145). In addition, time below range (glycaemia <54 mg/dL) was higher in the sulphonylurea group (0.22% versus 0.00%; p=0.048) and high-dose sulphonylureas were associated with sustained episodes of severe hypoglycaemia (β=0.487; p=0.028).^59^

Difficulties were encountered in analysing the effect of sulphonylureas on GV, since most of the included studies compared sulphonylureas versus another oral anti-diabetic drug. An adequate level of evidence indicates that sulphonylureas tend to increase GV. We suggest the use of other oral anti-diabetic drugs.

### Sodium–glucose cotransporter 2 inhibitors

Sodium–glucose cotransporter 2 (SGLT2) inhibitors inhibit a transport protein thereby increasing urinary glucose excretion, which, in turn, lowers glycaemia.^60^ SGLT2 inhibitors are useful in T2D as combination therapy with other oral anti-diabetic drugs and insulins to decrease GV and improve TIR.^60^ Additionally, SGLT2 inhibitors play an important role in increasing β cell mass and decreasing insulin doses required to achieve control.^60^

Henry et al. compared the impact of dapagliflozin versus placebo on the 24h glycaemic profile in adults with poor glycaemic control who were being treated with stable doses of insulin (≥30 U/day) or metformin (≥1,500 mg/day).^61^ An 18.2 mg/dL reduction in MBG, an increase in TIR of69.6%, and a reduction in GV was observed in the dapagliflozin group.^61^

SGLT2 inhibitors improve TIR and serum glucose levels without increasing episodes of hypoglycaemia,^24^ as suggested by Luo et al.^60^ When evaluating the effect of adding a dapaglifozin to insulin glargine and oral anti-diabetic therapy, there was a significant decrease in HbA1c, FBG, MBG, MAGE and hyperglycaemic excursions, together with an increase in TIR and a decrease in insulin doses required, without a statistically significant increase in hypoglycaemia episodes.^60^ A randomized controlled trial studying dapaglifozin as add-on therapy to insulin concluded that dapaglifozin reduced glucose levels but not glucose variability.^62^

There is controversial evidence that dapaglifozin improves GV, and there is insufficient evidence in order to make a conclusive statement in regards to SGLT2 inhibitors as a group.

### Glucagon-like peptide 1 agonists

A better understanding of the incretin system has allowed development of new therapies in T2D, such as GLP-1 receptor agonists, which stimulate the release of insulin in the post-prandial period.^48^ Endogenous GLP-1 is released into the bloodstream and rapidly degraded by the enzyme DPP4, therefore a molecule with similar action and longer half-l ife was developed.^48^ In addition to its insulinotropic effects, GLP-1 agonists significantly reduce body weight,^63–65^ which is fundamental considering 90% of patients with T2D are overweight or obese.^66^ There are currently several GLP-1 agonists available, which are divided into two categories: GLP-1 mimetics (natides) and receptor analogues (glutides). Glutides include drugs such as dulaglutide, semaglutide and liraglutide, which are administered subcutaneously.^48^

In a group of 68 patients with newly diagnosed T2D and a body mass index of 25-35 kg/m^2^, Li et al. evaluated the effect of insulin + liraglutide versus insulin monotherapy on GV, measured by the flash system.^67^ They observed a significant decrease in GV according to SD, CV and MAGE, and in oxidative stress markers in the liraglutide combination therapy group.^67^ Probstfield et al. obtained similar results in 102 patients with high cardiovascular risk, comparing insulin alone versus combination therapy of insulin and exenatide, with an improvement in mean CV (-2.4 versus 0.4; p=0.047).^68^ A meta-analysis assessing the efficacy of liraglutide on GV evaluated SD, MAGE and other parameters, and concluded that liraglutide was associated with lower GV (MAGE: I^2^=92%, p<0.01; Z=11.91, p<0.01; mean difference =-2.78, 95% CI -3.24 to -2.32; and SD: I^2^=93%, p<0.01; Z=3.62, p<0.01; standardized mean difference =-1.77, 95% CI -2.73 to -0.81).^69^

When comparing the addition of exenatide or placebo to metformin, Frías et al. observed that exenatide once weekly, compared with placebo, reduced MAGE (change from baseline: -15.12 mg/dL versus 2.88 mg/dL, respectively) and SD (change from baseline: -6.30 mg/dL versus 0.72 mg/dL, respectively).^70^ The exenatide group remained euglycaemic for longer (TIR 77% versus placebo 58%) with less time in hyperglycemia when compared with the placebo group (time in hyperglycemia of 22% versus 48%, respectively) and a similar time in hypoglycaemia (0.7% versus 0.3%, respectively).^70^ Umpierrez et al. compared the addition of lixisenatide versus placebo to basal insulin therapy.^71^ In 1,198 patients, there was a statistically significant reduction in SD in the lixisenatide group versus the placebo group (81.45 mg/dL versus 68.13 mg/dL).^71^ Moreover, when evaluating exenatide versus insulin glargine, McCall et al. identified a statistically significant decrease in mean daily risk (exenatide 16.33 ± 0.45 versus glargine 18.54 ± 0.49; p=0.001) with exenatide, mean daily risk being a parameter derived from glycaemic self-monitoring that quantifies the risk of glycaemic excursions.^72^ However, a randomized controlled study in China of patients treated with metformin, who additionally received exenatide or insulin glargine, reported a similar efficacy between these two drugs, though weight reduction was greater in the exenatide group.^73^ Regarding patients with cardiovascular comorbidities, Olmo-Garcia et al. compared insulin glargine with liraglutide (with insulin aspart as a corrective), finding a statistically significant decrease in GV determined by CV (liraglutide 20.98% versus glargine 25.48%) and SD (liraglutide 25.48 versus glargine 34.37) in the liraglutide group.^74^

In essence, the literature currently supports the use of GLP-1 agonists for controlling GV in T2D, being superior to the active comparator in most cases, in addition to benefits in weight reduction and cardiovascular risk.^75,76^

### α-Glucosidase inhibitors

α-Glucosidase inhibitors impede the absorption of carbohydrates from the small intestine by competitive inhibition. α-Glucosidase inhibitors delay digestion and absorption of carbohydrates, resulting in a slower and less pronounced rise in post-prandial glycaemia.^77^ They are not associated with hypoglycaemia.^78^

When comparing acarbose with metformin in patients with T2D using premixed insulin for 12 weeks, a significant improvement in GV indices was observed in both groups.^79^ However, the acarbose group showed a more pronounced change from baseline in CV (26.3% versus 11.9%, respectively; p=0.022), MAGE (40.5% versus 25.2%, respectively; p=0.007) and SD (38.6% versus 30.1%, respectively; p=0.041) than the metformin group.^79^ In another study, patients inadequately controlled with metformin + vildagliptin were randomized to either placebo or acarbose as add-on therapy, and assessed by CGM.^80^ MBG was 20 mg/ dL lower in the acarbose group (p<0.05), particularly during the postprandial period. AUC >180 mg/dL was 40% lower in the acarbose group, time >180 mg/dL was significantly higher in the placebo group (31% versus 8%, respectively; p<0.01) and MAGE was 20 mg/dL lower in the acarbose group.^80^

Acarbose improves GV. Additionally, combining an α-glucosidase inhibitor to decrease post-prandial glycaemia, and metformin to reduce FBG, may have a complementary effect. Existing literature supports the use of α-glucosidase inhibitors for reducing Hb1Ac, weight^81–83^ and risk of major adverse cardiovascular events.^84,85^ However, there is limited evidence that evaluates GV through indices previously mentioned in this article, suggesting that future studies should use these indicies to accurately observe effects on GV and compare pharmacological treatment options.

## Conclusion

It is clear that GV affects important goals of diabetes control (Hb1Ac, weight and hypoglycaemia), as well as the outcomes associated with glycaemic excursions. Glycaemic control in patients who are treated with insulin therefore must go beyond the classic control of glycaemic goals, and ensure the lowest GV. In T2D, concomitant non-insulin therapy may offer an alternative to mitigate the variability caused by insulin in susceptible patients. Based on the evidence presented in this review, it becomes evident that sulphonylureas play a deleterious role in terms of GV, while metformin appears to have a neutral effect. Among the options mentioned above, DPP4 inhibitors, GLP-1 agonists and SGLT2 inhibitors demonstrate promising effectiveness in managing GV. *Table 1* illustrates the main differences and impact on variability between these oral antidiabetic drugs. Based on currently available information, establishing a relevant role for any specific group is challenging, identifying a gap in our understanding of GV and its treatment. highlighting a gap in our understanding of GV and its treatment.

**Table 1:** Summary of impact of oral anti-diabetic drugs on glycaemic variability

Pharmacological group	Anti-diabetic action	Mechanism of action	Route of administration	Impact on variability	Literature findings
Insulins	Hypoglycaemic	Binding of the insulin receptor	Subcutaneous, intravenous	Increases	Supports the use of 2nd-generation basal-l ike insulins, such as insulin degludec-100 and insulin glargine-300, as they provide stable and prolonged glycaemic control.^39^ Furthermore, they significantly reduce GV, as evidenced by a decrease in MAGE from 74.9 mg/dL (95% CI, 60.3–97.7) to 64.8 mg/dL (95% CI, 52.0–78.2) (p<0.05) when comparing from insulin degludec + DPP4 inhibitor to insulin degludec + liraglutide, and reduce risk of hypoglycaemia.^36^
Biguanides	Anti-hyperglycaemic	Mitochondrial, intracellular anti-gluconeogenic effects, decreases insulin resistance	Oral	Neutral	No comparisons with placebo were identified due to the widespread use of metformin as a standard treatment in most glycaemic control protocols. The research reviewed did not show any statistically significant differences in GV measured by MAGE and SD.
Sulphonylureas	Hypoglycaemic	Secretagogue	Oral	Increases	Increase GV and hypoglycaemia. Therefore, it is recommended to consider other oral anti-diabetic drugs for glycaemic control.^57–59^
DPP4 inhibitors	Anti-hyperglycaemic	Incretins	Oral	Decreases	Significantly reduce GV (MAGE from 93.8 ± 38.0 mg/dL to 70.8 ± 19.2 mg/dL after treatment; p=0.046) with an adequate level of evidence.^54^
GLP-1 receptor agonists	Anti-hyperglycaemic	Incretins	Subcutaneous	Decreases	Supports their use for T2D control in terms of GV as exenatide once weekly, compared with placebo, reduced MAGE (change from baseline: -15.12 mg/dL versus 2.88 mg/dL, respectively) and SD (change from baseline: -6.30 mg/dL versus 0.72 mg/dL, respectively).^70^ They outperform active comparators in most cases and offer additional advantages such as weight loss and lower cardiovascular risk.^76^
SGLT2 inhibitors	Anti-hyperglycaemic	Glycosurics	Oral	Decreases	There is controversial evidence that dapaglifozin improves GV, and there is insufficient evidence in order to make a conclusive statement in regards to SGLT2 inhibitors as a group.^60–62^
α-Glucosidase inhibitors	Anti-hyperglycaemic	Delayed carbohydrate digestion and absorption	Oral	Decreases	Despite the limited amount of evidence regarding their impact on GV, current research endorses their use due to their effectiveness in reducing GV, Hb1Ac, weight and risk of major adverse cardiovascular events.^81,82,84^

*DPP4 = dipeptidyl peptidase-4; GLP-1 = glucagon-like peptide 1; GV = glycaemic variability; HbA1c = glycated haemoglobin; MAGE = mean amplitude of glycaemic excursion; SD = standard deviation; SGLT2 = sodium–glucose cotransporter-2; T2D = type 2 diabetes mellitus.*
